# Spatial transcriptomic characterization of the pathologic niche in a patient with pulmonary crystal-storing histiocytosis

**DOI:** 10.1016/j.gendis.2025.101904

**Published:** 2025-10-27

**Authors:** Yao Chen, Zhining Huang, Gaoxiang Wang, Mingsheng Wu, Xiao Chen, Xiaohui Sun, Lei Gao, Mingran Xie

**Affiliations:** a Department of Pathology, The First Affiliated Hospital of USTC, Division of Life Sciences and Medicine, University of Science and Technology of China, Hefei, Anhui 230001, China; b Department of Thoracic Surgery, The First Affiliated Hospital of USTC, Division of Life Sciences and Medicine, University of Science and Technology of China, Hefei, Anhui 230001, China

Crystal-storing histiocytosis (CSH) is a rare disease characterized by an accumulation of crystalline inclusions in the cytoplasm of histiocytes. These inclusions are derived from monoclonal immunoglobulin (Ig) deposition.^1^ The most frequently affected organs are the bone marrow and kidney; the lung is also an affected organ due to an imaging feature identical to that of lung cancer. CSH is usually an indirect sign of malignant diseases. As a result, most patients are concurrently diagnosed with neoplastic diseases such as multiple myeloma.^2^ Therefore, more in-depth investigations are urgently required to clarify the underlying mechanisms of CSH. The histological characterizations of CSH have been defined; however, the cellular and molecular features of CSH remain unclear.

Here, a 61-year-old male patient admitted with a left lower lobe lung nodule was diagnosed as pulmonary CSH (PCSH) after surgery; a spatial transcriptomic profile was then constructed. By integrating with healthy human lung samples,^3^ we defined the cellular composition and the expression patterns of light chains and immunoglobulin isotypes in the pathologic niche (Fig. 1A).

**Figure 1:**
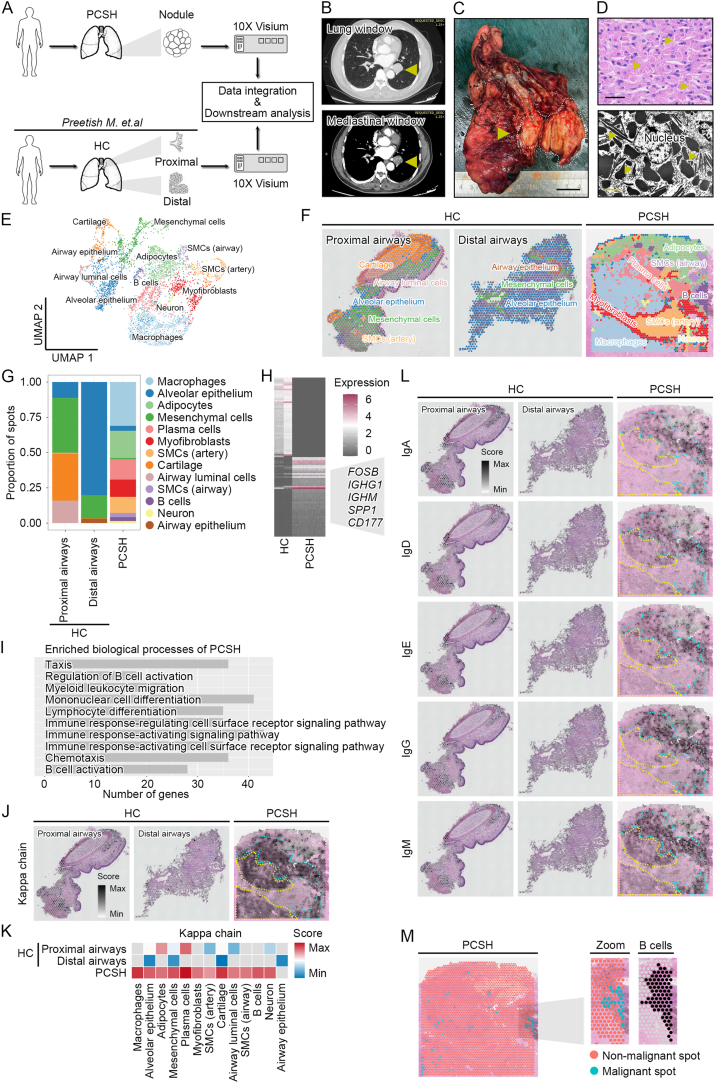
A spatial transcriptomic landscape of the human pulmonary crystal-storing histiocytosis (PCSH). **(A)** Schematic of the overall study design. HC, healthy control. **(B)** Chest CT scan results showing a 3.2-cm central left lower lobe lung nodule. CT, computed tomography. Arrowheads indicate the lung nodule. **(C)** Photograph of the resected left lower lobe. Scale bar, 2.0 cm. The arrowhead indicates the lung nodule. **(D)** Representative HE and TEM images of lung nodule sections. Arrowheads indicate histiocytes with intra-cytoplasmic rectangular and rhomboid crystals. Scale bar, 50 μm for the HE image; scale bar, 2 μm for the TEM image. Two-dimensional UMAP **(E)** and in situ **(F)** visualization of ST spot clusters obtained from the proximal airways, distal airways, and PCSH lung nodule. Spots are colored according to the cell types/structures. ST, spatial transcriptomic; UMAP, uniform manifold approximation and projection; SMCs, smooth muscle cells. **(G)** The proportion of cell types/structures across tissue sections. **(H)** The heatmap showing DEGs significantly expressed in PCSH. DEGs, differentially expressed genes. **(I)** Bar plot showing the top enriched biological process terms of the significantly up-regulated DEGs in PCSH. Spatial distribution map **(J)** and heatmap **(K)** of the genes encoding the components of the Kappa light chains. The Cyan frame indicates the plasma cell region; the yellow frame indicates the macrophage region. **(L)** Spatial distribution maps of the gene sets encoding the components of the Ig subtypes. The Cyan frame indicates the plasma cell region; the yellow frame indicates the macrophage region. **(M)** Annotation of malignant spots predicted by the Cancer-Finder algorithm. Right panel, in situ overlap between malignant spots and the B cell region.

The chest CT results revealed a 3.2-cm central left lower lobe lung nodule (Fig. 1B). Given the homogeneous solid appearance of this nodule, the patient did not receive any therapeutic interventions. After completing the preoperative assessments, this patient then received a sleeve lobectomy with bronchoplasty (Fig. 1C). The histological and transmission electron microscope (TEM) results showed that numerous aggregated histiocytes were filled with rectangular crystal-like structures (Fig. 1D; Fig. S1A). As expected, an extensive accumulation of macrophages was observed in PCSH lung nodule sections, as evidenced by the extensive expression of two canonical markers, CD68 and CD163 (Fig. S1B). We also found that the Kappa light chain was highly expressed (Fig. S1B); distinguished markers negatively expressed in PCSH were undetected (Fig. S1B). Collectively, we present a rare case of PCSH with a primary symptom of a lung nodule.

We then generated a transcriptome-wide spatial profile from PCSH tissue sections. Datasets generated from healthy human lungs were integrated with our spatial data using a defined Seurat workflow (Fig. S2).^4^ Unsupervised clustering of spots generated 13 distinct clusters based on the expression of canonical marker genes (Fig. 1E; Fig. S3). By referring to spatial annotations reported previously, the cellular proportion and identity of these cell types/structures were successfully annotated (Fig. 1F; Fig. S4). As expected, the distal airways consisted of alveolar epithelium. However, the original cell types/structures were replaced with a large number of adoptive immune cells, including histiocytes (also known as macrophages), plasma cells, and B cells (Fig. 1G). The lesion area of CSH is characterized by infiltrated histiocytes with crystals accumulated.^2^ However, our findings indicate that histiocytes are only a small subset; the remaining site is mainly composed of Ig-producing plasma cells and their naive form B cells. These results uncover the cellular heterogeneity of PCSH.

To explore the potential transcriptional changes of PCSH, we performed a pseudobulk differential expression analysis (Fig. 1H). Functional enrichment analysis revealed that PCSH-specific up-regulated genes were enriched in biological processes such as mononuclear cell differentiation and B cell activation (Fig. 1I). CSH is defined by an accumulation of immunoglobulin crystals produced by plasma cells.^5^ These results indicate that broad activation of B cell-mediated adaptive immunity occurs in the diseased tissues of PCSH and that this kind of activation in the mononuclear phagocytic cell system may function critically during the progression of CSH. We next sought to quantitatively evaluate the expression and distribution of their encoding genes at the spatial level by adopting a gene set-based approach. We found that genes encoding the components of the Kappa light chain were highly expressed in a restricted region consisting of macrophages, plasma cells, and B cells (Fig. 1J and K; Fig. S5). Scoring spots with genes encoding the components of Ig isotypes also revealed their distinct distribution patterns. The synthesis activity of the five Ig isotypes was highly active in plasma and B cells, and IgM was the most predominant isotype (Fig. 1L; Fig. S6). Moreover, the upstream regulatory signals were also activated in plasma and B cells (Fig. S7). These findings indicate that Ig synthesis, especially IgM, is isotype-restricted to the PCSH lesion area. Ig accumulation is supposed to be attributed to their resistance to lysosome degradation. A structural alteration in the amino acid sequence endows immunoglobulins with a hydrophobic nature.^5^ In contrast, we found that the transcripts of the Kappa chain are predominantly expressed, while the expression of Lambda is very limited. It is thus speculated that the difference in light chain accumulation is due to their expression variance but not degradation.

Patients with CSH are generally diagnosed with a neoplastic B cell disorder; PCSH is supposed to be a non-malignant condition, and the adjuvant therapy is unnecessary.^1^^,^^2^ However, whether malignant cells are present in the lesion area remains unclear. To address this issue, Cancer-Finder, a domain generalization-based deep learning algorithm, was adopted to infer the potential malignant spots. The majority of spots were allocated as non-malignant; however, some spots within the B cell-infiltrated region were identified as malignant (Fig. 1M). Therefore, both systematic examinations and long-term follow-up are highly recommended for PCSH patients.

In conclusion, we uncovered the cellular heterogeneity of PCSH and defined that the lesion area is massively infiltrated with histiocytes and Ig-producing plasma cells. In addition, while the Kappa chain is identified as the major subtype of light chain, the expression of Ig isotypes is also clarified as regionally heterogenous. However, due to the low incidence rate, CSH is generally reported in the form of a single case, and subsequent studies are delayed. The histological diagnosis of CSH is ambiguous during the surgery resulting in a delay in acquiring valuable tissue or blood samples. Together, multi-omics platform-based investigation of more cases is thus required and helpful in dissecting the pathogenesis of CSH.

## CRediT authorship contribution statement

**Yao Chen:** Writing – original draft, Investigation, Formal analysis, Data curation. **Zhining Huang:** Writing – original draft, Methodology, Investigation, Formal analysis, Data curation. **Gaoxiang Wang:** Methodology. **Mingsheng Wu:** Methodology. **Xiao Chen:** Methodology. **Xiaohui Sun:** Methodology. **Lei Gao:** Writing – review & editing, Validation, Supervision, Project administration, Funding acquisition, Conceptualization. **Mingran Xie:** Writing – review & editing, Supervision, Project administration, Funding acquisition, Conceptualization.

## Ethics declaration

All procedures followed were in accordance with the ethical standards of the responsible committee on human experimentation (institutional and national) and with the Helsinki Declaration of 1975, as revised in 2008. The human PCSH lung nodule sample was obtained with an approval by the Ethics Committee of The First Affiliated Hospital of University of Science and Technology of China (2024-RE-194).

## Data availability

The data that support the findings of this study are available from the corresponding authors upon reasonable request. The data reported in this paper have been deposited in the OMIX, China National Center for Bioinformation/Beijing Institute of Genomics, Chinese Academy of Sciences (https://ngdc.cncb.ac.cn/omix: accession no. OMIX011887). A spatial transcriptomic dataset of the healthy human lung consisting of proximal and distal airways was retrieved from the GEO under accession number GSE178361. The present study does not generate any new code. The code used in the present study, performed in R and Python, is provided in the Supplementary data.

## Funding

This work was supported by the 10.13039/501100001809National Natural Science Foundation of China (No.82100266, 81973643) and the Natural Science Research Project of Anhui Education Committee (China) (No. 2024AH052055).

## Conflict of interests

The authors declare that they have no conflict of interests.
