# Supplementary material for: Spatial transcriptomic characterization of the pathologic niche in a patient with pulmonary crystal-storing histiocytosis
Source: Genes Dis. 2025 Oct 27;13(3):101904. doi: 10.1016/j.gendis.2025.101904 (PMC12828807; doi:10.1016/j.gendis.2025.101904)
Supplement: Multimedia component 1 [file mmc1.docx]

**Materials and methods**

**Histology**

For rapid H&E staining during the surgery, the PCSH lung nodule sample was embedded in O.C.T. compound (4583, Sakura, USA) and sectioned. For regular H&E staining, the PCSH lung nodule sample was fixed with 10% neutral buffered formalin, embedded in paraffin, sectioned, and stained with H&E reagents. Immunohistochemistry staining was automatically performed with the BenchMark ULTRA PLUS system (Roche, USA). Primary antibodies used in the present study included CD68 (KP-1, M00101, Roche, USA), Kappa light chain (CH15, ZM-0160, Roche, USA), Cytokeratin (AE1/AE3, ZM-0069, ZSGB-BIO, China), Myogenin (EP162, ZA-0592, ZSGB-BIO, China), CD163 (MRQ-26, V0003369, Roche, USA), Langerin (12D6, ZM-0385, ZSGB-BIO, China), and Ki-67 (12D6, K13163, Roche, USA). Images were automatically processed using IHC profiler in the ImageJ software. Tissues were assigned a score based on the staining intensity (0 = no staining; 1 = low positive; 2 = positive; 3 = high positive).

**Transmission electronic microscopy**

The formalin-fixed, paraffin-embedded (FFPE) PCSH lung nodule section was treated to remove the paraffin (deparaffinization), fix in 1% osmium tetroxide, embed in Pon812 epoxy resin, and section. The sample was then processed as previously reported;^1^ images were captured using an electron microscope (Tecnai F12, FEI, USA).

**Spatial transcriptomic data generation and processing**

Spatial transcriptomic data generation of the human PCSH lung nodule section was conducted by the LC-Bio Technology CO. Ltd. (Hangzhou, China) using the Visium Spatial Gene Expression for FFPE Reagent Kit (1000338, User Guide CG00407 Rev C, 10x Genomics). According to the user guide, the FFPE tissue section was subjected to deparaffinization, H&E staining, imaging, and decrosslinking. The sequencing library was then prepared. The library was pooled and sequenced on NovaSeq 6000 platform (Illumina). FastQ files were processed by using the Space Ranger (Version 2.0.0, 10x Genomics) pipeline. Sequence reads were mapped to the reference genome GRCh38 (human).

**Spatial transcriptomic data integration**

In order to integrate the spatial transcriptomic data of the human PCSH and healthy human lung consisted of proximal and distal airways, an acknowledged Seurat (Version 5.1.0) workflow was adopted with default parameters.^2^ In brief, loaded Seurat objects were pre-processed using the “SCTransform” function. The slices were then integrated with the “merge” function. Dimensional reduction and unsupervised clustering were achieved with the following steps including “RunPCA”, “FindNeighbors”, “FindClusters”, and “RunUMAP” functions. This integrated Seurat object was subjected to the downstream analysis unless mentioned.

**Calculation and visualization of the gene set score**

To visualize the expression and distribution of Ig subtypes and two light chains, several defined gene sets were downloaded from MSigDB.^3^ Enrichment scores were calculated with the “AddModuleScore” function with default parameters; results were visualized using the “SpatialFeaturePlot” function.

**Differential expression and functional enrichment analyses**

To identify the cluster or group-specific differentially expressed genes, the object was prepared with the “PrepSCTFindMarkers” function; the processed object was then analyzed using the “FindAllMarkers” function. Heat map was generated by using the “DoHeatmap” function with top 50 differentially expressed genes extracted. Gene ontology enrichment analysis was performed with the “enrichGO” function in the clusterProfiler package.^4^

**Malignant state annotation**

To identify the potential malignant region in spatial transcriptomic data of the human PCSH, Cancer-Finder, a Python-based algorithm, was employed with default parameters.^5^ The annotated malignant state was visualized using the “SpatialDimPlot” function.

**References**

1. Huang J, Wang L, Shen Y, et al. CDC-like kinase 4 deficiency contributes to pathological cardiac hypertrophy by modulating NEXN phosphorylation. *Nat Commun.* 2022;13(1):4433.

2. Hao Y, Stuart T, Kowalski MH, et al. Dictionary learning for integrative, multimodal and scalable single-cell analysis. *Nat Biotechnol.* 2024;42(2):293-304.

3. Liberzon A, Birger C, Thorvaldsdóttir H, Ghandi M, Mesirov JP, Tamayo P. The Molecular Signatures Database (MSigDB) hallmark gene set collection. *Cell Syst.* 2015;1(6):417-425.

4. Yu G, Wang LG, Han Y, He QY. clusterProfiler: an R package for comparing biological themes among gene clusters. *Omics.* 2012;16(5):284-287.

5. Zhong Z, Hou J, Yao Z, et al. Domain generalization enables general cancer cell annotation in single-cell and spatial transcriptomics. *Nat Commun.* 2024;15(1):1929.


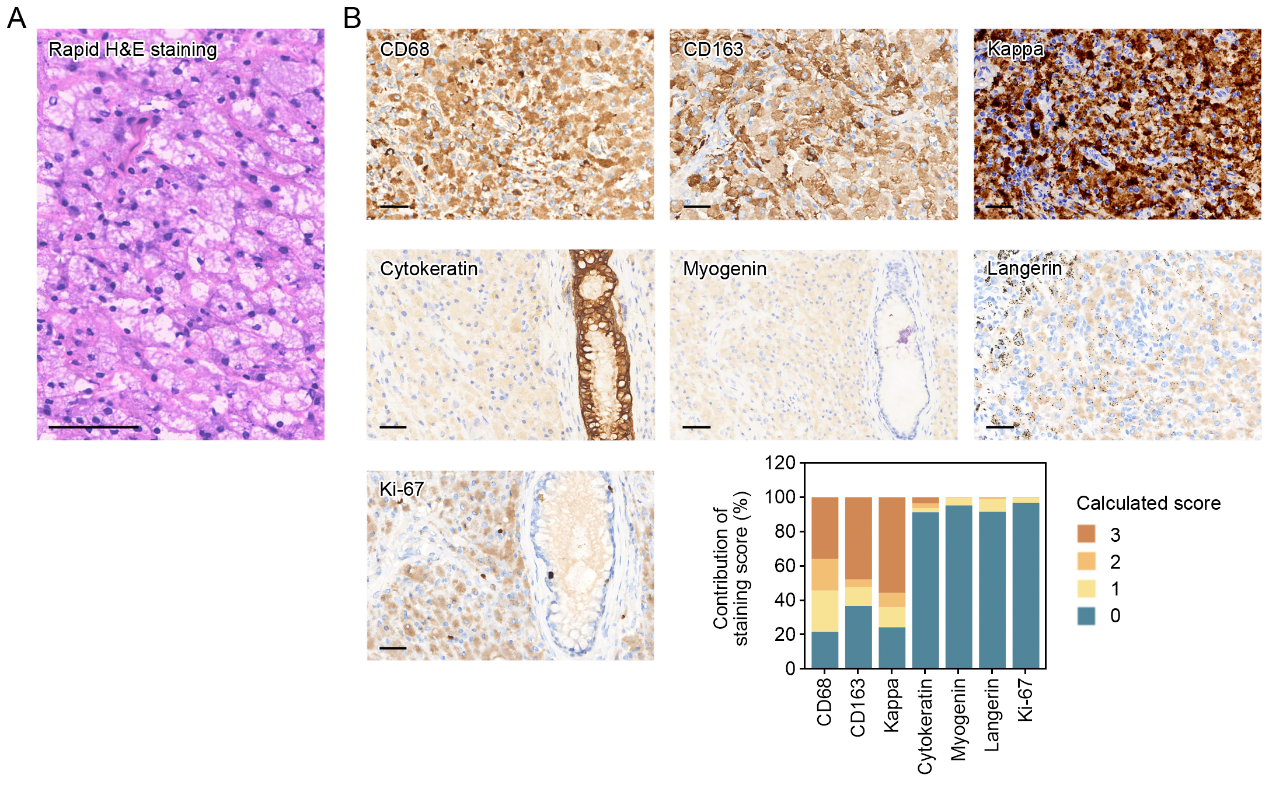


**Figure S1** Pathological diagnosis of PCSH. (**A**) Representative image of rapid H&E staining result of lung nodule section during the surgery. Scale bar, 50 µm. (**B**) Representative images of immunohistochemistry analysis for CD68, CD163, Kappa light chain, Cytokeratin, Myogenin, Langerin, and Ki-67. Scale bar, 100 µm. Immunohistochemistry staining was automatically quantified and scored on a scale of 0 (negative staining) to 3 (highly positive staining).


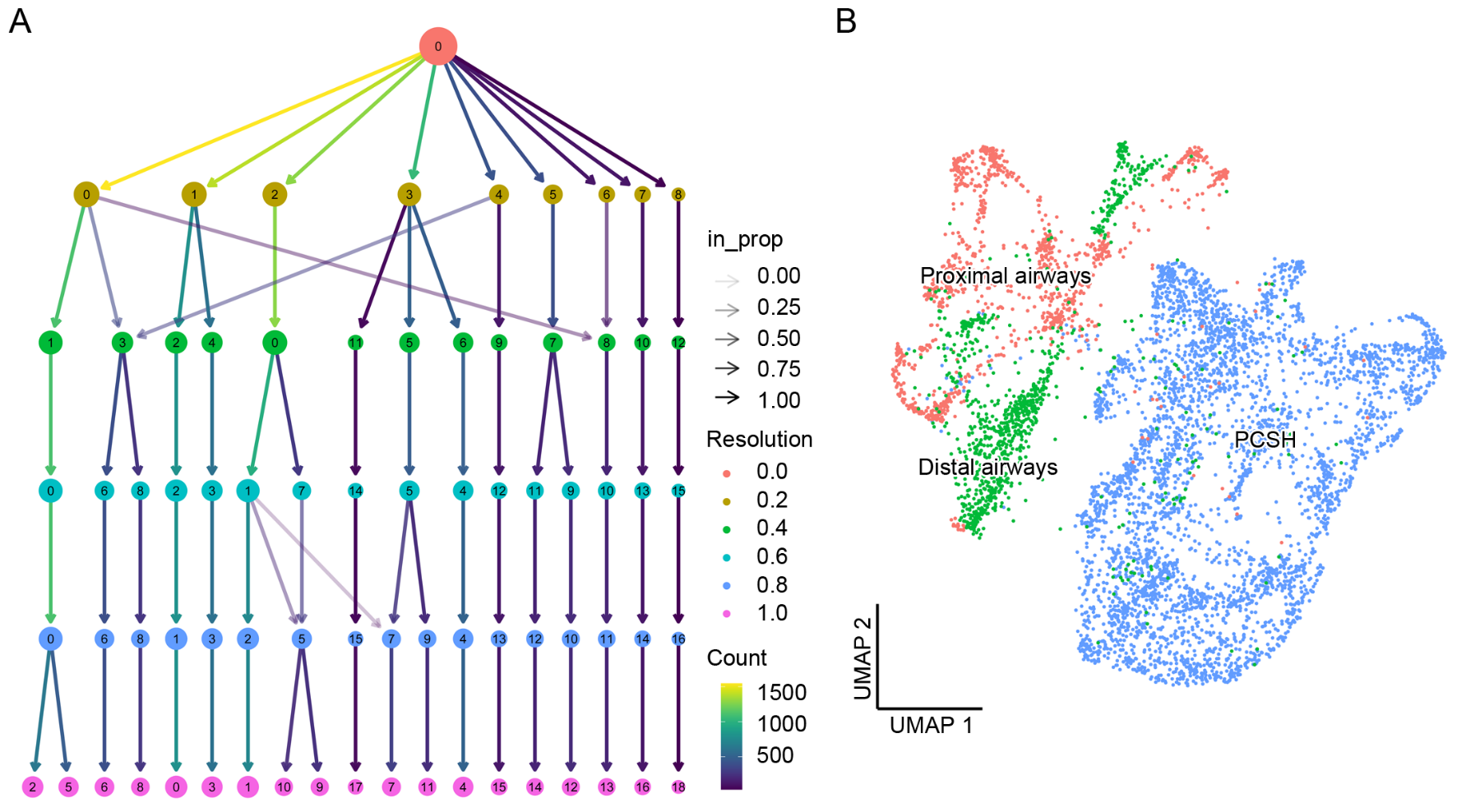


**Figure S2** Quality control of the integration workflow. (**A**) Clustering tree analysis at different resolutions. (**B**) Two-dimensional UMAP visualization of integrated ST spots obtained from proximal airways, distal airways, and PCSH lung nodule. Spots are colored by samples. ST, spatial transcriptomic.


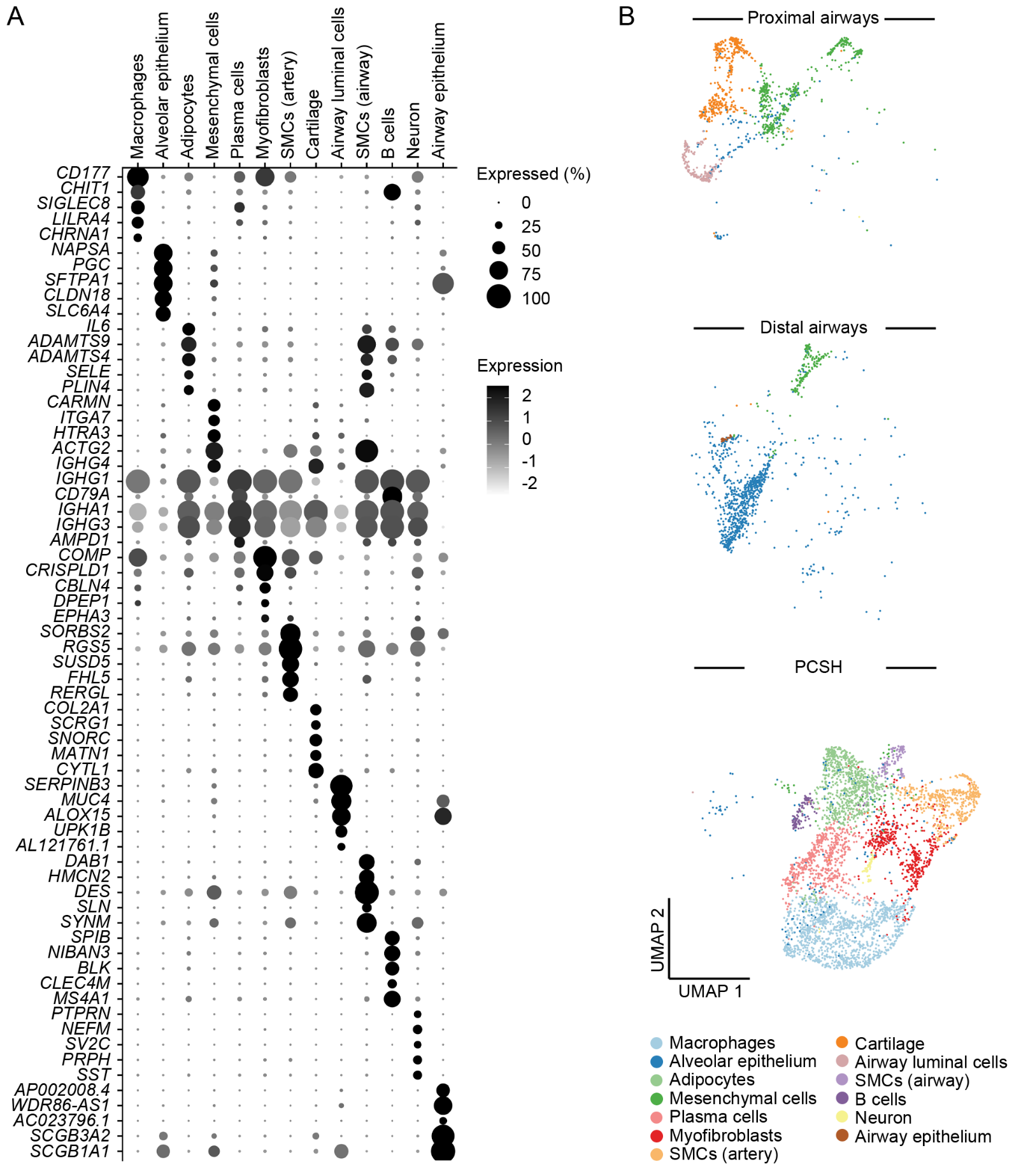


**Figure S3** Annotations of cell types/structures. (**A**) Dot plot showing the expression of marker genes in each cell type/structure in the integrated ST data. (**B**) Two-dimensional UMAP visualization of ST spot clusters as split by samples. Spots are colored by cell types/structures.


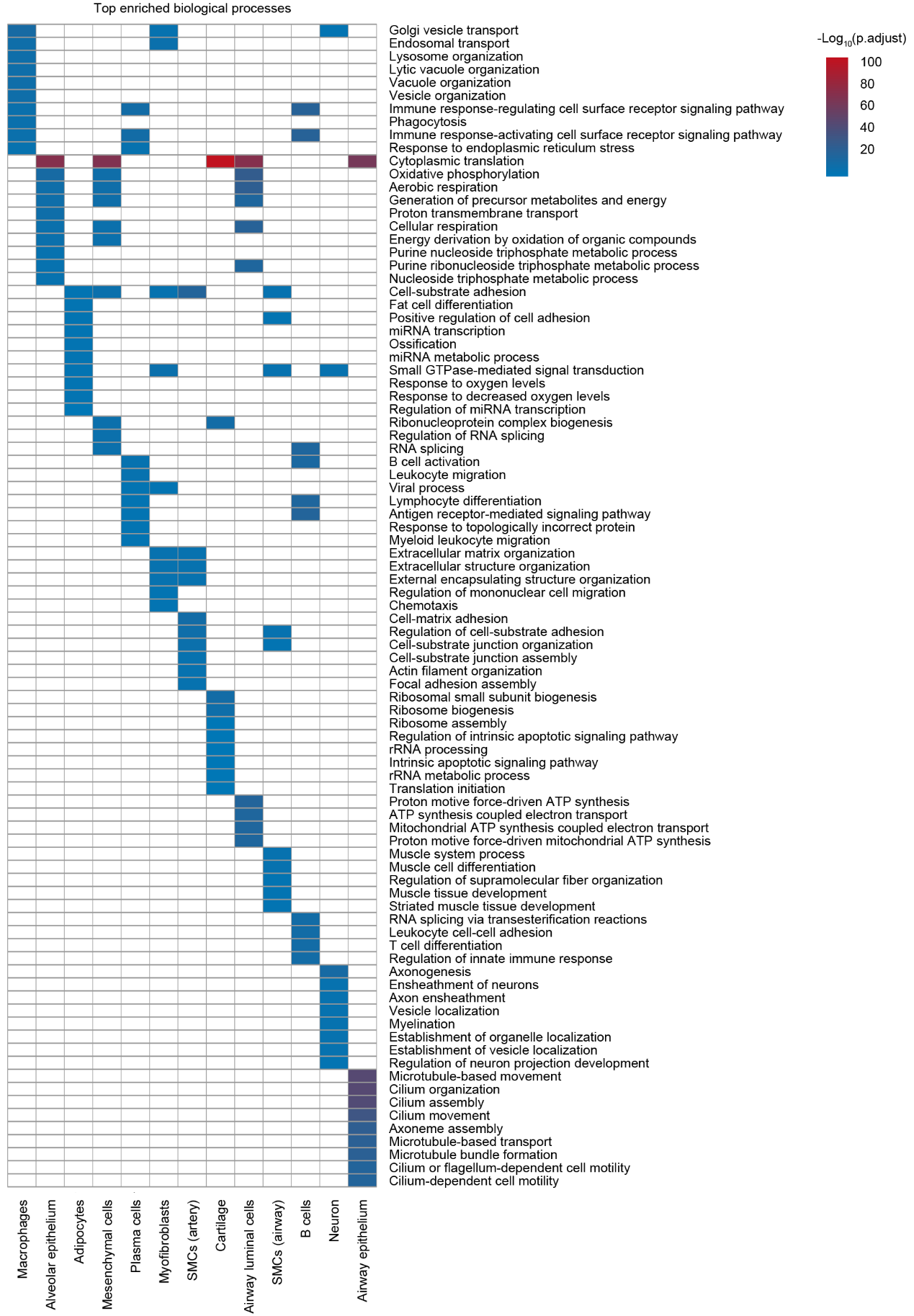


**Figure S4** Top enriched biological process terms of differentially expressed genes for each cell type/structure.


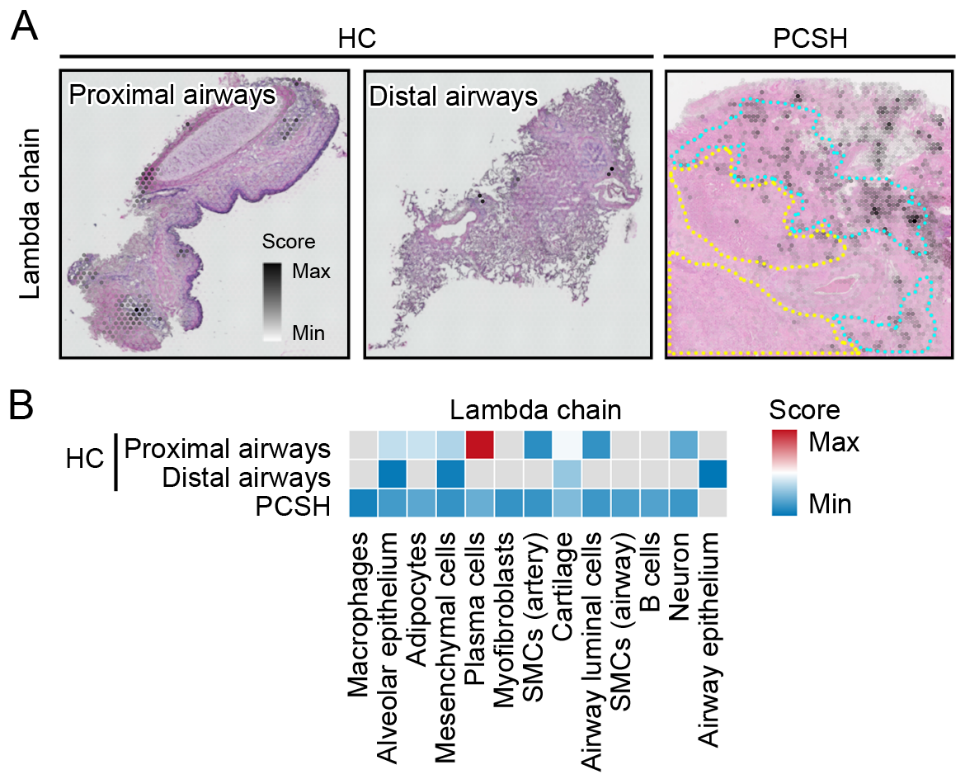


**Figure S5** Spatial analysis of Lambda light chain. Spatial distribution map (**A**) and heatmap (**B**) of the genes encoding the components of Lambda light chain. Cyan frame indicates plasma cell region; yellow frame indicates macrophage region.


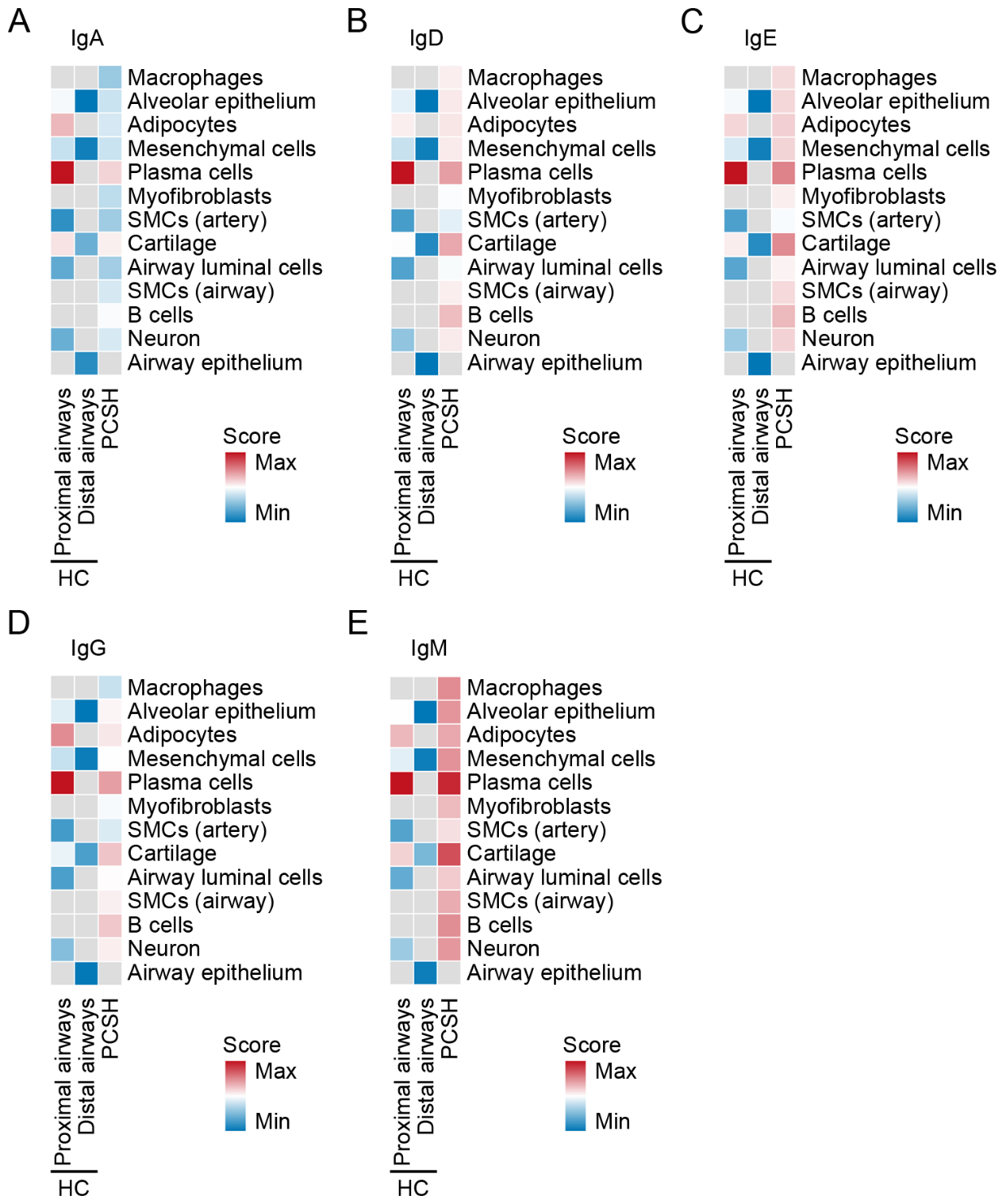


**Figure S6** Quantitative analysis of Ig subtype. Heatmaps showing the scores of gene sets encoding the components of IgA (**A**), IgD (**B**), IgE (**C**), IgG (**D**), and IgM (**E**).


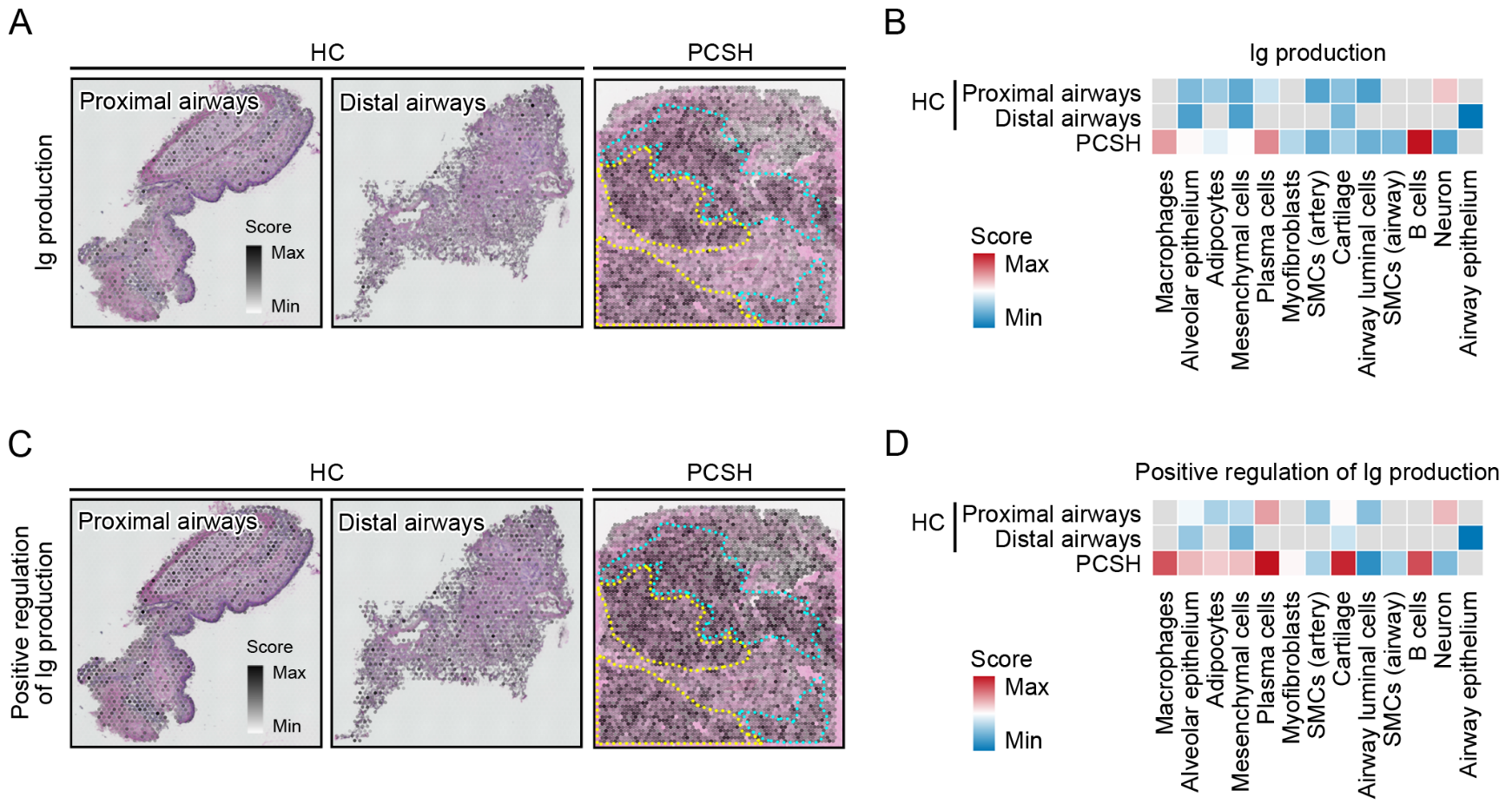


**Figure S7** Spatial analysis of upstream regulons of Ig production. Spatial distribution map (**A**) and heatmap (**B**) of the gene set encoding the proteins promoting Ig production. Cyan frame indicates plasma cell region; yellow frame indicates macrophage region. Spatial distribution map (**C**) and heatmap (**D**) of the gene set encoding the proteins involved in the positive regulation of Ig production process.
